# Effects of Blanching and Natural Convection Solar Drying on Quality Characteristics of Red Pepper (*Capsicum annuum* L.)

**DOI:** 10.1155/2017/4656814

**Published:** 2017-09-10

**Authors:** James Owusu-Kwarteng, Francis K. K. Kori, Fortune Akabanda

**Affiliations:** ^1^Department of Applied Biology, Faculty of Applied Sciences, University for Development Studies, P.O. Box 24, Navrongo, Ghana; ^2^Department of Applied Physics, Faculty of Applied Sciences, University for Development Studies, P.O. Box 24, Navrongo, Ghana

## Abstract

The objective of this work was to determine the effects of blanching and two drying methods, open-sun drying and natural convection solar drying, on the quality characteristics of red pepper. A 2 × 3 factorial design with experimental factors as 2 drying methods (open-sun drying and use of solar dryer) and 3 levels of pepper blanching (unblanched, blanched in plain water, and blanched in 2% NaCl) was conducted. Dried pepper samples were analysed for chemical composition, microbial load, and consumer sensory acceptability. Blanching of pepper in 2% NaCl solution followed by drying in a natural convection solar dryer reduced drying time by 15 hours. Similarly, a combination of blanching and drying in the solar dryer improved microbial quality of dried pepper. However, blanching and drying processes resulted in reduction in nutrients such as vitamin C and minerals content of pepper. Blanching followed by drying in natural convection solar dryer had the highest consumer acceptability scores for colour and overall acceptability, while texture and aroma were not significantly (*p* > 0.05) affected by the different treatments. Therefore, natural convection solar dryer can be used to dry pepper with acceptable microbial and sensory qualities, as an alternative to open-sun drying.

## 1. Introduction

Red pepper (*Capsicum annuum* L.) is used as a spice or major ingredient in dishes around worldwide and perhaps considered the first spice to have been used by man with archaeological evidence of pepper and other fossil foods dating back to 6000 years old [1]. Red pepper is generally known to be cholesterol free, have low sodium and caloric contents, and serve as good source of vitamins A and C [2]. Additionally, red pepper is known to possess antimicrobial activity [3] and reduces the risk of diseases such as arthritis, cancer, and diabetes [4–8]. In food processing, red pepper is also used as colouring and flavouring agent in sauces, soups, pickles, and pizzas [9].

Ghana contributes about 1% of world pepper production and was ranked the eleventh largest producer of pepper in the world and second in Africa with an estimated total production of 88,000 metric tons [10]. Like other fresh fruits and vegetables, fresh pepper is a perishable produce and deteriorates within a few days after harvest without proper storage or preservation measures. The perishable nature of pepper can lead to economic losses which is further aggravated by storage and marketing problems and lack of appropriate processing technologies [11]. To prevent losses due to postharvest deterioration, dehydration is one important method often adopted for preservation and value addition to perishable agricultural products through moisture control. The major goal in drying fruits and vegetables such as pepper is to reduce the moisture content to desirable levels, usually 5–10%, which allows for safe storage over an extended period of time [12].

Unlike many industrialized countries where mechanized drying of fruits and vegetables is practiced, traditional open-sun drying of fruits and vegetables is a common practice in developing countries. However, traditional open-sun drying of fruits and vegetables can be time-consuming and less hygienic. Although modern mechanized drying of food commodities is faster than open-sun drying and uses much less land space, the equipment cost as well as the continued recurring cost of fuel or energy to operate these systems is very high. Therefore, small-scale farmers and other players in the pepper value chain in rural communities in Ghana resort to the traditional open-sun drying method of preservation which results in low-quality products. As an alternative to mechanized and open-sun drying methods, solar dryers are being investigated and used for drying various fruits and vegetables [12], especially at geographic locations where there is enough sunshine during the harvest season [13]. Further advantages of solar drying over conventional open sun drying are the improvement in hygienic quality of the dried products, safe moisture content, colour and taste, and the protection of produce from rain, dust, and insects [14].

To obtain dried pepper with the best organoleptic and nutritional qualities, pretreatment such as blanching may be applied prior to drying [15–17]. Blanching is considered a pretreatment or unit operation prior to freezing, canning, or drying in which fruits or vegetables are heated for the purpose of inactivating enzymes; modifying texture; preserving colour, flavour, and nutritional value. Therefore, the objective of this work was to determine the effects of aquathermal blanching and natural convection solar drying methods on the quality characteristics of pepper.

## 2. Materials and Methods

### 2.1. Design and Fabrication of Natural Convection Solar Dryer

**Figure 1:**
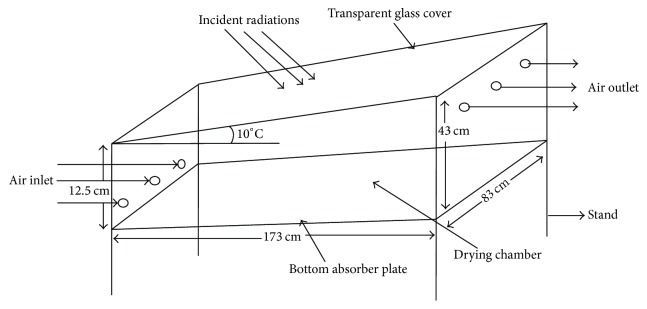
Schematic diagram of solar dryer.

A direct natural convection solar dryer used in this study was designed and fabricated as shown in Figure 1. It was constructed mainly with wood on the sides and a transparent glass (166 cm × 76 cm) covering the top inclined at an angle of 10° to face the equator. A metal plate, painted black to improve absorption, was fitted at the bottom of the box with cardboard (insulator) beneath it. The inner side walls of the box were lined with aluminum foil for the reflection of radiation back to the interior space of box. Three inlet and outlet vents were created to allow air convectional current take place.

Further details and measurements of the solar collector are shown in Table 1. The constructed direct solar drying system was installed in an open place at the Navrongo Campus of the University for Development Studies. The drier was positioned to face the equator to maximize the collection of solar radiation throughout the day. Navrongo is located at 10.8940°N, 1.0921°W.

### 2.2. Experimental Design

A 2 × 3 factorial design with experimental factors as two pepper drying methods (open-sun drying and use of solar dryer) and 3 levels of pepper blanching (unblanched, blanched in plain water, and blanched in 2% NaCl) were conducted. The samples were analysed for their chemical composition including moisture, crude protein, ash, and fibre contents [18]. Additionally, mineral content, microbial counts, and consumer sensory analysis of dried pepper samples were carried out following standard methods.

#### 2.2.1. Sample Treatments

Fresh* Capsicum annuum *L. (red pepper) were purchased from the local open market in Navrongo. The pepper was first washed thoroughly under running tap water and then blanched at 93°C in plain or in 2% NaCl solution for 4 min. Prior to drying experiments, the initial moisture content of fresh untreated pepper was determined by drying in an oven set at 120°C until constant mass was observed. The initial and final masses of the red pepper samples were recorded with an electronic balance. Initial moisture content of red pepper was found to be about 75% on wet basis.

#### 2.2.2. Drying of Pepper

Differently treated pepper samples were loaded onto wire-mesh trays at 5 kg/m^2^ and dried by suspending them on weighing balance in the drying chamber of the direct solar dryer and in the open sun simultaneously. The samples were spread evenly in a single layer on the stainless-steel wire-mesh trays and kept inside the drying chamber of the natural convection solar dryer or in the open sun. The drying experiments were carried out in the month of May, 2016, at University for Development Studies, Navrongo Campus, Ghana. Each experiment started at 09:00 a.m. and continued until 5:00 p.m. daily. Relative humidity during the experimental period ranged between 55% and 65%. During the experimental period, ambient temperature, temperatures within the dryer, and the absorber plate temperatures were measured at 1 h intervals. Additionally, the mass of pepper was measured at 1 h intervals throughout the experiments until a final moisture content of 5% (ideal for long-term storage of pepper) was achieved [12].

#### 2.2.3. Chemical Analysis of Dried Pepper

Crude protein, fibre, and ash were determined following the procedures by AOAC methods 970.22, 985.29, and 972.15, respectively [18]. Mineral analyses were carried out using AOAC procedure of Atomic Absorption Spectrophotometer (AAS) [18]. Briefly, one (1) ml aliquots of the digest from pepper sample were used to determine Ca and Fe contents of pepper using Spectra AA 220FS Spectrophotometer (Varian Co., Mulgrave, Australia) with an acetylene flame.

#### 2.2.4. Microbiological Analysis

Serial 10-fold dilutions were made by weighing 10 g of dried pepper samples into 90 ml of Butterfield's phosphate buffer (Hardy Diagnostics, CA). Appropriate dilutions were surface-plated onto Tryptic Soy Agar (TSA; Difco) supplemented with cycloheximide (50 mg/l) to inhibit the growth of mold and onto CHROMagar ECC plates and incubated at 37°C for 24 h. Following incubation, all visible colonies on TSA were enumerated as Aerobic Mesophilic count (AMC), and all pink (coliforms) and blue (presumptive* E. coli*) colonies on CHROMagar ECC plates were enumerated. Microbial counts were reported as CFU/g of pepper sample.

#### 2.2.5. Consumer Sensory Evaluation of Dried Pepper

Consumer sensory quality of the dried pepper was carried out to assess attributes including colour, texture, aroma, and overall acceptability by 65 volunteered untrained panelists drawn from the Faculty of Applied Sciences of the University for Development Studies. The panelist were adults between the ages of 18 and 40 years who are familiar with whole dried pepper. The panelist independently, in separate sensory evaluation booths, assessed the various products for sensory qualities using a nine-point hedonic scale with 1, 5, and 9 representing “dislike extremely,” “neither like nor dislike,” and “like extremely,” respectively [19]. All products were presented to the panelists randomly and placed side by side, with each panelist receiving two rounds of each product.

### 2.3. Statistical Analysis

All experiments were carried out in triplicate and values are presented as means with standard deviations (SD) where applicable. Data obtained were subjected to analysis of variance (ANOVA) and Least Significant Difference (LSD) was used to separate means at *p* < 0.05 using the MINITAB statistical software package (MINITAB Inc. Release 14 for Windows, 2004).

## 3. Results and Discussion

### 3.1. Ambient and Solar Dryer Temperatures

**Figure 2:**
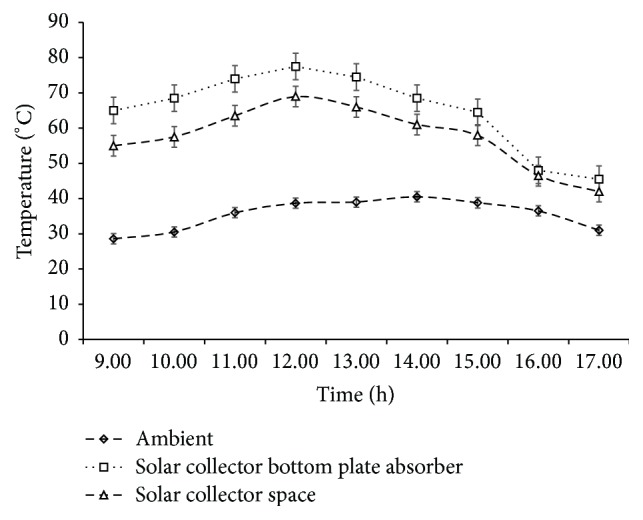
Ambient and collector temperatures on sunny hours of the day during drying experiments.

Variations in ambient temperature and temperatures in the drying chamber of solar dryer and absorber plate during the experimental period are shown in Figure 2. The average ambient temperature increased from 28.6°C at 9:00 GMT to 40.5°C at 14:00 GMT and decreased thereafter to 31°C at 17:00 GMT. In a similar trend, temperature of the drying chamber within the solar dryer increased from an average 55.0°C at 9:00 to 69°C at 12:00 GMT and decreased thereafter to average of 42°C at 17:00 GMT. The highest average temperatures of drying chamber and bottom absorber plate of the solar dryer, 69.0°C and 77.5°C, respectively, were attained at 12:00 GMT.

### 3.2. Drying Characteristics of Pepper

**Figure 3:**
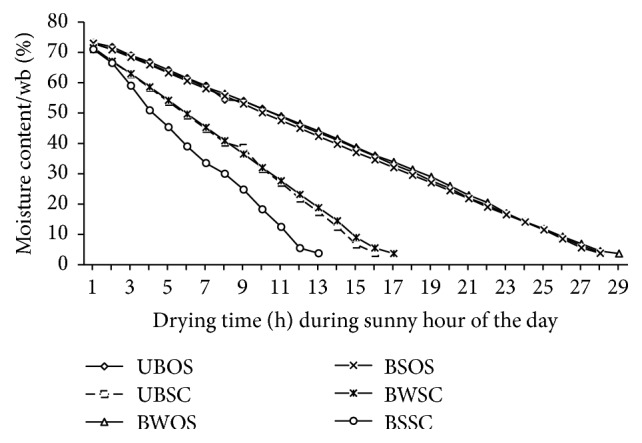
Moisture content of pepper during drying in open sun and in solar dryer. Drying was stopped when moisture content reduced to 5%. UBOS: unblanched open-sun drying; UBSC: unblanched solar collector drying; BWOS: blanched in plain water open-sun drying; BSOS: blanched in 2% NaCl solution open-sun drying; BWSC: blanched in plain water solar collector drying; BSSC: blanched in 2% NaCl solution solar collector drying.

The reduction in moisture contents of pepper samples during drying in the open sun and in solar dryer is shown in Figure 3. Fresh pepper samples had an average moisture content of 75% prior to pretreatment and drying. Pepper samples that were blanched in 2% NaCl solution and subsequently dried in the solar dryer had moisture content reduced to 5% after 13 h of drying. Pepper samples that were blanched in plain water and unblanched pepper attained moisture contents of 5% after drying in a period of 16 and 17 h, respectively, in solar dryer. On the other hand, all pepper samples that were dried in the open sun, irrespective of pretreatment, attained moisture contents of 5% after 28 h of drying. Thus, blanching in 2% NaCl followed by use of the natural convection solar dryer reduced the drying time for pepper by 15 hours. In general, average drying time for pepper samples that were dried in the natural convection solar dryer was significantly lower (*p* < 0.05) than pepper samples dried in the open sun. Blanching either in 2% NaCl solution or in water significantly (*p* < 0.05) improved drying rate of pepper (Figure 3). In general, drying pepper in the solar dryer reduced drying time by about 49–54% depending on pretreatment method applied to the pepper.

In a review of various types of solar drying systems for agricultural commodities, Fudholi et al. [20] reported that the moisture content of fresh chili decreased from 80% to 5% under solar drying in 48 h. Improvement in the drying rates of blanched pepper has previously been reported [5, 16, 21–23], and the observation has been attributed to possible rupturing of cell membrane making pepper tender and thus facilitating faster removal of moisture in the drying process [22].

### 3.3. Effect of Blanching and Drying Method on Nutrient Composition of Pepper

The nutrient composition of pepper following blanching treatments and drying is shown in Table 2. Total proteins, ash, and fibre contents of fresh pepper were 2.8 ± 0.04, 2.6 ± 0.10, and 4.8 ± 1.00 (g/100 g), respectively, while vitamin C, calcium (Ca), and iron (Fe) contents were 175.6 ± 1.5, 18.5 ± 0.9, and 2.1 ± 0.10 (mg/100 g), respectively. Generally, crude proteins and fibre contents were not significantly affected by the blanching treatments and drying methods. However, crude ash content significantly reduced following blanching and drying. Similarly, vitamin C, Ca, and Fe contents reduced significantly (*p* < 0.05) after blanching treatments and drying (Table 2).

Generally, vegetables and fruits serve as good sources of energy, minerals, and vitamins. However, during dehydration processes, changes in nutritional quality of heat sensitive vitamins and other nutrients occur [12, 24]. It has previously been reported that significant losses in the content of vitamin C, minerals, and polyphenols in vegetables occur after aquathermal blanching [25, 26], an observation which may be due to their sensitivity to heat and/or leaching of these compounds in water [24, 27]. However, steam blanching was reported to retain higher amounts of vitamin C in spinach compared with hot water blanching [28]. The present results show that neither blanching in NaCl solution nor plain water could significantly retain the vitamin C and mineral contents of pepper as there were significant losses in these nutrients after blanching and drying.

### 3.4. Microbial Load in Dried Pepper

**Figure 4:**
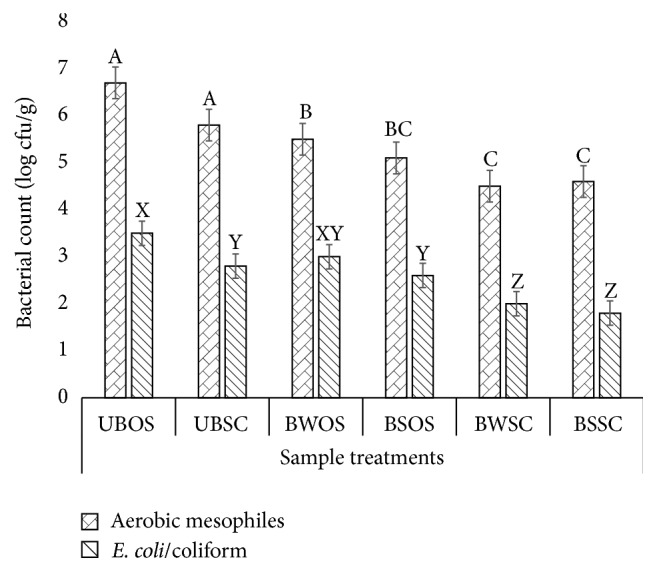
Mean aerobic mesophilic and* E. coli*/coliform counts in dried pepper. For AMC or ECC, means (±SD) with different letters are significantly different (*p* < 0.05). UBOS: unblanched open-sun drying; UBSC: unblanched solar collector drying; BWOS: blanched in plain water open-sun drying; BSOS: blanched in 2% NaCl solution open-sun drying; BWSC: blanched in plain water solar collector drying; BSSC: blanched in 2% NaCl solution solar collector drying.

Mean aerobic mesophilic counts (AMC) and* E. coli*/coliform (ECC) counts in dried pepper are shown in Figure 4. Aerobic mesophilic counts ranged between 4.6 ± 1.2 and 6.7 ± 0.8 cfu/g, while ECC counts ranged between 1.8 ± 0.5 and 3.5 ± 1.0 cfu/g of dried pepper samples.

Generally, pepper samples that were blanched and dried in solar collector had significantly (*p* < 0.05) lower microbial load when compared to samples that were dried in the open sun with or without blanching (Figure 4). Thus, a combination of blanching and the solar collector drying processes can significantly reduce the microbial load in dried pepper. Through blanching and drying to control moisture levels (water activity, *a*_*w*_) in pepper, microbial load can be controlled although spores may not be killed. These may bring microbial load of dried pepper to allowable safe limits while supporting long shelf life of these products.

### 3.5. Consumer Sensory Evaluation of Dried Pepper

Consumer sensory evaluation of the differently treated and dried pepper samples assessed using a 9-point hedonic scale is shown in Table 3. Consumer evaluation of dried pepper covered sensory characteristics such as colour, aroma, texture, and overall acceptability. Results of consumer evaluation generally showed no significant difference (*p* > 0.05) in aroma and texture between the different pepper samples. However, significant differences (*p* < 0.05) were observed in colour and overall acceptability between samples. For colour, pepper samples that were blanched either in plain water or in 2% NaCl solution and subsequently dried using the solar dryer scored significantly higher (7.0), whereas pepper samples that were not blanched and subsequently dried in the open sun scored significantly (*p* < 0.05) lower (3.8). For overall acceptability, the highest score of 7.1 was attained for pepper samples that were blanched in 2% NaCl solution and subsequently dried in the solar dryer, indicating the panelist likeness for the product.

It has been suggested that inactivating the enzymes responsible for browning (polyphenoloxidase, lipoxygenase, and peroxidase), during blanching improves both colour and flavour of vegetables [29]. However, if not carefully managed, blanching may also be accompanied by a reduction in sensory and nutrient qualities in many foods, principally due to Maillard reaction [30].

## 4. Conclusions

Blanching of pepper in 2% NaCl solution followed by drying in a natural convection solar dryer reduced drying time by 15 hours when compared to drying in the open sun which takes at least 28 hours. Similarly, a combination of blanching and drying in the solar dryer improved microbial quality of the dried pepper. However, the blanching and drying processes resulted in reduction of nutrients such as vitamin C and minerals contents. Consumer sensory analysis showed that blanching, followed by drying in natural convection solar dryer, had the highest scores for colour and overall acceptability, while texture and aroma were not significantly (*p* > 0.05) affected by the various treatments. Therefore, natural convection solar dryer has the potential for commercial small-medium scale industrial application for drying pepper with acceptable microbial and sensory qualities, especially in developing rural communities where equipment cost as well as recurring cost of fuel or energy to operate mechanical dryers can prove to be high. This in turn would ensure reduction in postharvest losses of pepper and thus better economic returns for farmers and processors.

## Figures and Tables

**Table 1:** Details of solar collector assembly material.

Parameter	Value/description
Collector area	1.44 m2 (0.83 m × 1.73 m)
Absorber plate	Galvanized steel 0.67 mm thick
Absorber plate surface treatment	Black paint coating
Glazing	Transparent glass of surface area 1.26 m^2^ and 5 mm thick
Bottom insulation	Cardboard 20 mm thickness
Inside reflector	Aluminium foil of 0.5 mm thickness
Casing	Hard wood of 2.5 cm thick
Collector slope	10°

**Table 2:** Nutrient composition of dried pepper^*∗*^.

Sample	Protein (g/100 g)	Ash (g/100 g)	Fibre (g/100 g)	Vitamin C (mg/100 g)	Ca (mg/100 g)	Fe (mg/100 g)
Fresh pepper	2.8 ± 0.04^a^	2.6 ± 0.07^a^	4.8 ± 1.00^a^	175.6 ± 1.5^a^	18.5 ± 0.9^a^	2.1 ± 0.10^a^
UBOS	2.7 ± 0.10^a^	1.5 ± 0.05^b^	4.7 ± 0.81^a^	103.0 ± 0.8^b^	13.0 ± 1.1^b^	0.9 ± 0.05^b^
UBSC	2.8 ± 0.06^a^	1.4 ± 0.01^bc^	4.8 ± 0.50^a^	99.5 ± 1.2^c^	12.3 ± 0.8^cb^	1.1 ± 0.08^b^
BWOS	2.7 ± 0.05^a^	1.2 ± 0.08^cd^	4.8 ± 1.02^a^	101.0 ± 0.9^bc^	14.5 ± 0.9^bd^	0.8 ± 0.06^cb^
BS0S	2.8 ± 0.12^a^	1.5 ± 0.06^b^	4.6 ± 0.65^a^	100.3 ± 1.5^bc^	14.3 ± 1.0^bd^	0.7 ± 0.07^cd^
BWSC	2.7 ± 0.08^a^	1.1 ± 0.10^d^	4.7 ± 0.50^a^	98.8 ± 1.1^c^	15.0 ± 0.5^bd^	0.9 ± 0.12^b^
BSSC	2.7 ± 0.05^a^	1.3 ± 0.05^bc^	4.8 ± 0.50^a^	109.9 ± 0.8^bc^	15.2 ± 1.2^bd^	0.7 ± 0.05^cd^

^*∗*^Means (±SD) values with different letters as superscripts in a column are significantly different (*p* < 0.05). UBOS: unblanched open-sun drying; UBSC: unblanched solar collector drying; BWOS: blanched in plain water open-sun drying; BSOS: blanched in 2% NaCl solution open-sun drying; BWSC: blanched in plain water solar collector drying; BSSC: blanched in 2% NaCl solution solar collector drying.

**Table 3:** Consumer sensory evaluation of dried pepper.

Sensory attribute	BWOS	BSOS	UBOS	BWSC	BSSC	UBSC
Colour	5.8 ± 1.9^a^	5.9 ± 2.0^a^	3.8 ± 2.1^b^	6.8 ± 2.0^c^	7.0 ± 1.7^c^	4.1 ± 1.9^b^
Aroma	6.4 ± 2.2^a^	6.1 ± 1.9^a^	6.3 ± 1.9^a^	6.0 ± 1.8^a^	6.3 ± 1.6^a^	6.2 ± 1.8^a^
Texture	5.9 ± 1.8^a^	5.7 ± 1.5^a^	6.1 ± 2.0^a^	5.7 ± 1.7^a^	5.8 ± 1.7^a^	6.1 ± 1.7^a^
Overall acceptability	6.2 ± 1.4^a^	6.4 ± 1.2^a^	4.6 ± 1.7^b^	6.9 ± 1.7^c^	7.1 ± 1.9^c^	4.8 ± 2.0^b^

UBOS: unblanched dried in open sun; UBSC: unblanched dried in solar collector; BWOS: blanched in plain water and dried in open sun; BSOS: blanched in 2% salt solution and dried in open sun; BWSC: blanched in plain water and dried in solar collector; BSSC: blanched in 2% salt solution and dried in solar collector. Values with same superscript in a row are not significantly different (*p* > 0.05).
